# A prognostic model for failure and worsening after lumbar microdiscectomy: a multicenter study from the Norwegian Registry for Spine Surgery

**DOI:** 10.1007/s00701-021-04859-3

**Published:** 2021-07-10

**Authors:** David A. T. Werner, Margreth Grotle, Milada Cvancarova Småstuen, Sasha Gulati, Øystein P. Nygaard, Øyvind Salvesen, Tor Ingebrigtsen, Tore K. Solberg

**Affiliations:** 1 Department of Neurosurgery, University Hospital of Northern Norway, Tromsø, Norway; 2 Department of Clinical Medicine, Faculty of Health Sciences, UiT the Arctic University of Norway, Tromsø, Norway; 3 Faculty of Health Sciences, Oslo Metropolitan University, Oslo, Norway; 4 Communication Unit for Musculoskeletal Disorders (FORMI), Oslo University Hospital, Oslo, Norway; 5 The Norwegian Registry for Spine Surgery (NORspine), University Hospital of Northern Norway, Tromsø, Norway; 6 Department of Neurosurgery, St. Olav University Hospital, Trondheim, Norway; 7 Department of Neuromedicine and Movement Science, Norwegian University of Science and Technology, Trondheim, Norway

**Keywords:** Microdiscectomy, Outcome, PROM, Quality, ODI, Lumbar disc surgery

## Abstract

**Objective:**

To develop a prognostic model for failure and worsening 1 year after surgery for lumbar disc herniation.

**Methods:**

This multicenter cohort study included 11,081 patients operated with lumbar microdiscectomy, registered at the Norwegian Registry for Spine Surgery. Follow-up was 1 year. Uni- and multivariate logistic regression analyses were used to assess potential prognostic factors for previously defined cut-offs for failure and worsening on the Oswestry Disability Index scores 12 months after surgery. Since the cut-offs for failure and worsening are different for patients with low, moderate, and high baseline ODI scores, the multivariate analyses were run separately for these subgroups. Data were split into a training (70%) and a validation set (30%). The model was developed in the training set and tested in the validation set. A prediction (%) of an outcome was calculated for each patient in a risk matrix.

**Results:**

The prognostic model produced six risk matrices based on three baseline ODI ranges (low, medium, and high) and two outcomes (failure and worsening), each containing 7 to 11 prognostic factors. Model discrimination and calibration were acceptable. The estimated preoperative probabilities ranged from 3 to 94% for failure and from 1 to 72% for worsening in our validation cohort.

**Conclusion:**

We developed a prognostic model for failure and worsening 12 months after surgery for lumbar disc herniation. The model showed acceptable calibration and discrimination, and could be useful in assisting physicians and patients in clinical decision-making process prior to surgery.

## Background

Worldwide, low back pain is the leading cause for years lived with disability [14]. The most common indication for low back surgery is sciatica caused by lumbar disc herniation (LDH) [9]. The lifetime prevalence of sciatica in the general population has been reported between 12 and 27% [19]. If left untreated, most patients with LDH will have a favorable outcome. Surgery is typically offered to patients with persisting and/or intolerable leg pain with or without low back pain, or with severe limb or bowel/bladder paresis (cauda equina syndrome) [3, 28]. The majority of the operations are performed electively on relative indications.

Most clinical studies tend to focus on favorable outcomes after surgery based on mean improvements or success rates according to patient-reported outcome measures (PROMs) [2, 3, 20, 28, 37], and predictive models for such outcomes have been developed [22, 24, 25]. An efficient strategy for improving the quality and safety of the health service is to increase the focus on unfavorable outcomes [8, 35]. Although the majority of patients experience substantial improvements, up to 30–40% report non-successful outcomes [2, 12, 23, 38], and a large proportion of these cases cannot be classified as “failure” [6], indicating that non-success and failure are not interchangeable concepts.

The risk of a poor outcome is a frequent concern among patients being operated, especially the risk of getting worse, which indicates a harmful (adverse) treatment effect [32]. To enhance individualized risk prediction and prevention of unfavorable outcomes, we have previously defined benchmark criteria for both failure and worsening, based on frequently used PROMs [38]. A prediction model for unfavorable outcomes can be further developed into a risk calculator, which could enhance shared clinical decision-making and improve selection of patients prior to lumbar disc surgery.

The aim of this study was to develop a prognostic model calculating individual risk (%) for failure and worsening after surgery for lumbar disc herniation, based on a large cohort from the Norwegian registry for spine surgery (NORspine). Data from this large registry cohort, collected in daily surgical practice, would ensure high external validity, and thus clinical relevance.

## Material and methods

### Design

Multicentre observational study following the recommendations for reporting in observational studies, STROBE criteria [36], and the methodological framework proposed by the PROGRESS group [34].

### Study population and data collection

A total of 26,427 patients operated for degenerative disorders of the lumbar spine reported to the NORspine registry between January 1, 2007 and August 2, 2015 were screened for eligibility and followed for 12 months. The NORspine includes patients operated for degenerative disorders of the spinal column. It does not include patients with fractures, primary infections of the spine, or with spinal malignancies. Furthermore, it does not include children <16 years of age, as well as patients with known serious drug abuse or severe psychiatric disorders. For the purpose of this study, we included all patients who had a microscope or loupe assisted lumbar disc microdiscectomy for a magnetic resonance imaging (MRI) confirmed lumbar disc herniation. Both emergency and elective cases were registered. Patients diagnosed with lumbar spinal stenosis or spondylolisthesis, and those operated with more comprehensive decompression techniques including laminectomy, disc prosthesis or fusion procedures, were excluded.

The NORspine is a comprehensive clinical registry for quality control and research, covering 95% of public and private operating centers in Norway, with a completeness (proportion of operated patients reported to the registry) of 65% over the study period. It comprises a range of baseline data on known and potential predictors for different outcomes [27]. Participation in NORspine is not required for a patient to gain access to the health care, or to receive payment/reimbursement for a provider.

At admission for surgery (baseline), the patients completed a questionnaire on demographics, lifestyle issues, and the PROMs. During the hospital stay, the surgeon recorded data concerning diagnosis, treatment, and comorbidity on a standard registration form. Twelve months after surgery, a questionnaire identical to that used at baseline was distributed by regular mail. It was completed at home by the patients and returned to the central registry unit without involvement of the treating hospitals. One reminder with a new copy of the questionnaire was sent to those who did not respond.

Informed consent was obtained from all patients.

The NORspine registry protocol has been approved by the Data Protection Authority of Norway. This study was submitted to the regional ethical committee for medical research which categorized it as a clinical audit study (2015/1829/REK South-East Regional Health Authority).

### Outcomes

Failure and worsening were defined according to validated cut-offs on the Oswestry Disability Index (ODI) version 2.1a, which showed the highest accuracy identifying these outcomes when evaluated against the numeric rating scale for back pain, leg pain, and the EuroQol 5D (EQ-5D) [38]. The ODI contains ten questions about limitations of activities of daily living. Each item is rated from 0 to 5 and then transformed into a score ranging from 0 (none) to 100 (maximum pain-related disability) [4]. The ODI cut-offs have been determined according to an external anchor, the global perceived effect scale (GPE, 1–7): 1 “fully recovered,” 2 “much better,” 3 “somewhat better,” 4 “unchanged,” 5 “somewhat worse,” 6 “much worse,” 7 “worse than ever.” Failure corresponds to GPE range 4–7, and worsening to GPE range 6–7 [38, 39]. We have also shown that that both the ODI change score, as well as the final ODI score after 12 months are highly dependent on the preoperative ODI score [38, 39]. Therefore, we stratified our model according to the preoperative ODI score (percentiles). Failure was defined as an ODI raw score 12 months after lumbar microdiscectomy ≥18 (low baseline ODI group, < 25 percentile), ≥ 29 (medium baseline ODI group, 25 to 75 percentile), and ≥ 34 (high baseline ODI group, > 75^th^ percentile). Worsening was defined accordingly as an ODI raw score 12 months after lumbar discectomy ≥33 (low baseline ODI group), ≥ 47 (medium baseline ODI group), and ≥ 58 (high baseline ODI group) [38].

### Possible prognostic factors

We included prognostic factors, previously reported in the literature [10, 12, 15, 17, 18, 29]. Sociodemographic and anthropometric factors included were; gender, age > 60, obesity (body mass index, BMI ≥ 30), marital status (living alone yes/no), employment status (employed/unemployed), and low educational level (yes/no), i.e., less than 4 years of college/university education. Anxiety or depression was assessed by the item on the EuroQol-5D-3L questionnaire, (yes = “moderate” to “severe” problems, no = “no problems”). In Norway, public health insurance is compulsory; thus, no distinction was made between public or private insurance, or between public and private hospitals. A recent study has shown equivalent effectiveness of lumbar disc surgery between the public and private sector [21]. Patients were also asked if they had a pending or unresolved claim or litigation issue (yes/no) against (1) the Norwegian public welfare agency fund concerning permanent disability pension or (2) a compensation claim against private insurance companies or the public Norwegian System of Compensation to Patients. As shown in the tables, we also assessed other clinical parameters, including the baseline PROM scores, smoking, duration of symptoms, previous lumbar spine surgery, and use of analgesics [12, 15, 17, 18, 29].

### Statistical analyses

All statistical analyses were performed with the Statistical Package for the Social Sciences (SPSS, IBM Version 23.0) and R (Version 2.13.1.) To assess potential sources of selection bias among patients, baseline differences between respondents and non-respondents at 12 months of follow-up were evaluated using the Students *t*-test for continuous variables or chi-square test for pairs of categorical variables. The proportions of missing data were small, <10% for all the analyzed variables. No imputation of missing values was performed.

**Fig. 1:**
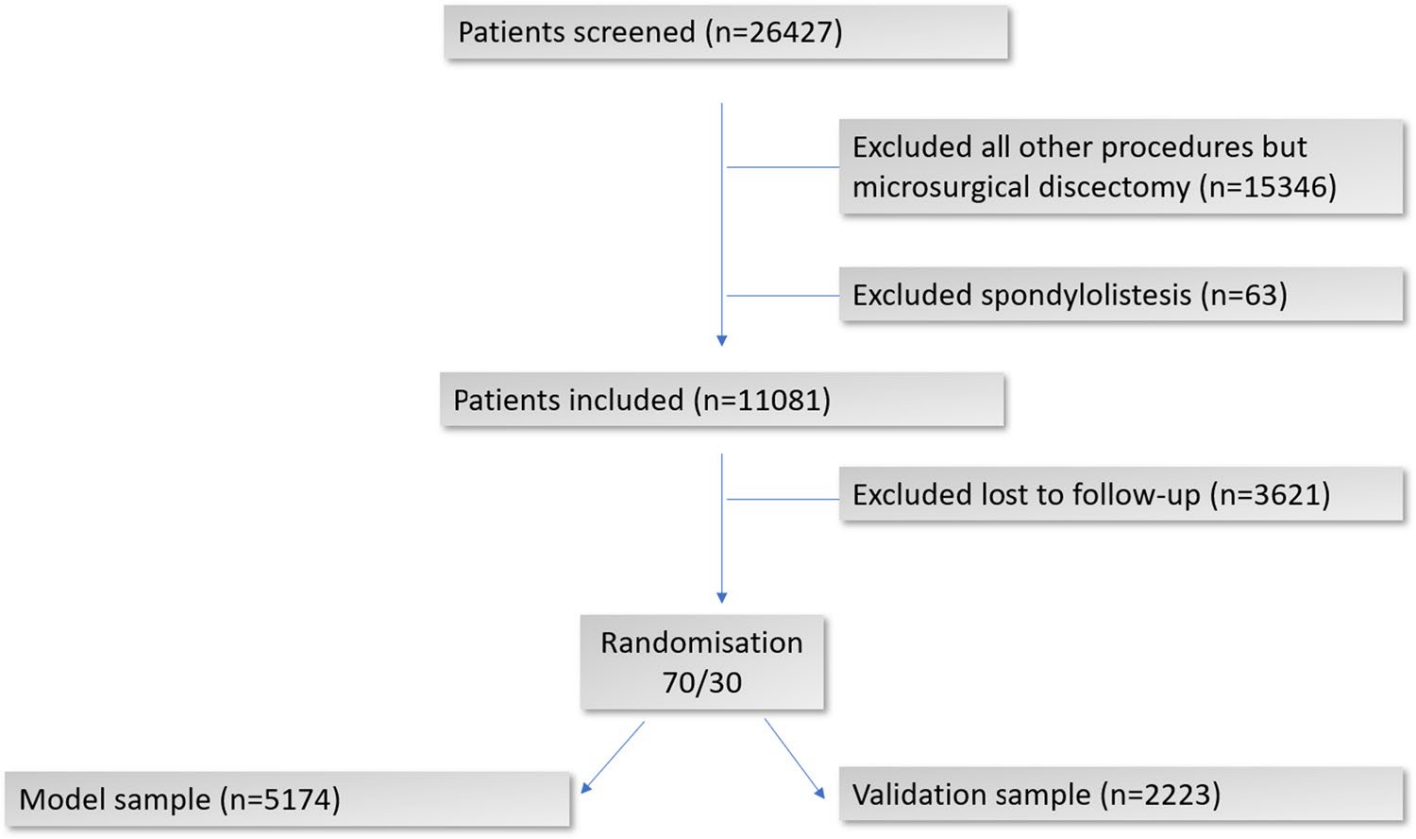
Flow diagram of patient enrollment, exclusion and allocation

Cases were selected for the training set (70%, *n* = 5741) and validation set (30%, *n* = 2218,) by the random sample function in SPSS (Fig. 1) [7]. The models were built using the training set, and then the final models were assessed in the validation set. Since the ODI threshold values for failure and worsening after 12 months depend on the preoperative ODI baseline score, we stratified the prediction model into the three ODI percentiles of “low” ODI baseline scores (<33), “medium” (33–58), and “high” (>58) for each outcome[38, 39].

### Training set

**Fig. 2:**
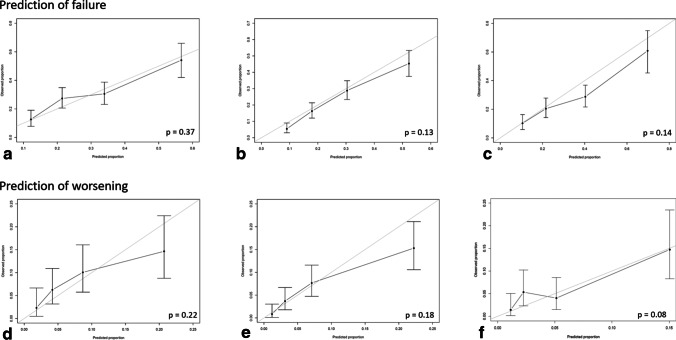
Model validation. Observed proportion of the outcome (with confidence interval) on the vertical axis against average predicted probability of the outcome on the horizontal axis. Each coordinate with whiskers represents one quartile of estimated probability and its 95% confidence interval, compared to the observed proportion of the predicted outcome. The p value from the chi square test for the coordinates vs the optimal prediction line is indicated in the lower right corner. A *p* value < 0.1 indicates significant deviation from the average predicted probability. ***a***, ***b****, ****c*** show prediction of failure for the three baseline invalidity groups (A: baseline ODI < 25^th^ percentile, B 25–75^th^ percentile, C > 75^th^ percentile). ***d***, ***e***, ***f*** show prediction of worsening for the three baseline invalidity groups (D: baseline ODI < 25^th^ percentile, E: 25^th^–75^th^ percentile, F: > 75^th^ percentile)

The outcomes failure versus no failure and worsening versus no worsening were modeled separately (Fig. 2).

Crude associations between each selected covariate and the outcome were assessed using univariate logistic regression. Variables that reached *p* < 0.1 in these analyses were entered into the multivariate analyses (binary logistic regression model). In a next step, variables that were no longer statistically significant (*p* < 0.05) were removed from the model using backward selection. We chose to include gender and age in all models, irrespectively of their statistical significance [31]. Continuous variables were dichotomized in order to be adapted into a risk matrix. Collinearity between possible predictors was assessed with Spearmans rho, with correlation coefficients (CC) >0.3 considered as weak, >0.5 as moderate, and > 0.7 as strong. Associations between outcomes and prognostic factors were expressed as odds ratios (OR) with a 95% confidence interval (CI). Regression coefficients from the final models were converted into probabilities for the risk matrix. Depending on the presence or absence of the risk factors, the matrix then calculated a probability for both failure and worsening for each patient.

### Validation set

For each model, calibration was assessed by dividing the sample into four prediction groups (quartiles) with increasing probabilities for failure and worsening. We then plotted the observed proportion for these outcomes against the average predicted probability, using a logistic regression model with the observed binary outcome as dependent and the log odds of the validated regression model as independent. Chi square test was used to assess difference between predicted coordinates and the optimal prediction line. Significant deviation, indicating over- or underestimation, was defined as *p*-values <0.1. Discrimination was assessed by the c-criterion (C), calculated as the area under the curve (AUC) in a receiver operating analysis (ROC), plotting predicted probability against failure and worsening. C values >0.6 were considered acceptable [31].

## Results

### Study population and data collection

We included 11,081 patients in the analyses. Of these, 3621 (32.7%) were lost to follow-up 12 months after surgery (Fig. 1). Baseline characteristics for the entire study population are shown in Table 1.

**Table 1:** Baseline characteristics including patient-reported outcome measures of respondents vs. non-respondents (lost to follow-up)

Characteristics	Respondent	Non-respondent	*P* value
	*n* = 7397 (67%)	*n* = 3621 (33%)	
Female	3097 (41.9)	1374 (38.1)	< 0.001
Age > 60	1403 (19)	307 (8.6)	< 0.001
Living alone	1642 (22.4)	1048 (29.3)	< 0.001
Non-native speaker	416 (5.6)	240 (6.7)	0.031
Low education^1^	2870 (39.1)	1168 (32.8)	< 0.001
Had leg pain	7156 (96.7)	3518 (97.7)	0.007
Leg pain > 12 months	1668 (23.8)	855 (25.5)	0.066
Back pain > 12 months	2441 (34.8)	1219 (36.0)	0.212
Operated for paresis	1542 (20.8)	651 (18.1)	0.001
Paresis < grade 4	529 (35.2)	195 (30.7)	0.046
Emergency surgery	757 (10.2)	350 (9.7)	0.417
Comorbidity^2^	1891 (29.1)	842 (26.9)	0.026
ASA^3^ grade > 2	408 (5.6)	152 (4.3)	0.004
Smoker	1935 (26.4)	1317 (37.0)	< 0.001
Obesity^4^	1236 (18.6)	735 (22.4)	< 0.001
Diabetes mellitus	236 (3.2)	95 (2.6)	0.123
Anxiety/depression^5^	3062 (42.1)	1608 (45.8)	< 0.001
Unresolved disability pension issue^6^	879 (12.3)	398 (11.3)	0.173
Unresolved insurance claim^7^	419 (5.8)	230 (6.5)	0.167
Previous surgery	1602 (21.7)	932 (25.9)	< 0.001
Previously operated > 2 times	72 (1.0)	53 (1.5)	0.026
PROMs	mean (SD)	mean (SD)	
ODI^8^	46.3 (19.2)	45.7 (18.6)	0.166
EQ-5D	0.27 (0.36)	0.25 (0.36)	0.125
NRS^9^ back pain	6.2 (2.5)	6.4 (2.4)	0.024
NRS leg pain	6.9 (2.2)	6.9 (2.2)	0.492

^1^Less than 4 years of college/university education. ^2^Rheumatoid arthritis, ankylosing spondylitis, other rheumatic disorder, hip arthrosis, knee arthrosis, chronic generalized musculoskeletal pain, chronic neurologic disorder, cerebrovascular disorder, heart disease, vascular disease, chronic lung disease, cancer, osteoporosis, hypertension, diabetes mellitus, other endocrine disorder. ^3^American Society of Anesthesiologists grade. ^4^Body mass index ≥ 30. ^5^EQ-5D 3L questionnaire; 5^th^ item, moderate to severe problems. ^6^Pending medical claim/litigation against the Norwegian public welfare agency fund concerning disability pension. ^7^Pending medical compensation claim/litigation against private insurance companies or the public Norwegian System of Compensation to Patients. ^8^Oswestry Disability Index, 0–100 (no-maximal disability). ^9^Numeric rating scale (0–10)

Mean age was 47.8 years (SD 13.61), and 42% of patients were females. Non-respondents at 12 months were younger, more likely to be men, had less severe comorbidity, and less severe limb paresis, but were more likely to be smokers, obese, anxious or depressed, and previously operated. There were no clinically relevant differences in baseline pain and disability (PROMS) between respondents and non-respondents. The amount (*n*, %) of missing data for the prognostic factors was low for age (6, 0.01), gender (none), non-native Norwegian speaker (19, 0) living alone (43, 0.01), smoking (76, 0.01), having low education (52, 0.01), BMI (522, 11.2), American Association of Anesthesiologists (ASA) grade > 2 (128, 1.8), unresolved disability pension issue (182, 3.4), unresolved insurance claim (171, 3.4), anxiety/depression (117, 1.6), duration of back pain >12 months (391, 5.6), back pain intensity (176, 2.4), and leg pain intensity (157, 2.2). Patient-reported outcomes by baseline ODI (percentiles) subgroups in the training and the validation sets are shown in table 5 (supplementary appendix). For the entire study population, a total of 1779 cases (24.1%) were classified as failed and 469 (6.3%) as worsened.

### Prognostic factors and outcomes

Tables 6 and 7 in the supplementary appendix show the results from the univariate analyses for all potential prognostic factors for failure and worsening, in both the training and validation sets. The results from the multivariate regression analyses for all three ODI baseline groups are shown in Table 2 (failure) and 3 (worsening). Duration of preoperative back pain was highly correlated (CC >0.7) with duration of preoperative leg pain. Duration of preoperative leg pain was consequently excluded from the model because of suspected multi-collinearity**.** Otherwise, all correlations between potential prognostic factors were low (CC ≤0.3).

**Table 2:** Results from the multiple regression model showing associations (odds ratio (OR) and 95% confidence intervals (CI)) between predictors and patient-reported “failure” (unchanged or worse, yes/no) of lumbar disc surgery, as defined by validated cut offs on the Oswestry Disability Index (ODI), split on subgroups with low, medium and high baseline ODI scores (percentiles). For all predictors, except age and gender, NS indicates statistical insignificance, *p* value > 0.05

	OR for failure by baseline ODI score^1^								
	Low ODI < 25^th^ percentile			Medium ODI 25^th^–75^th^ percentile			High ODI > 75^th^ percentile		
Predictor	OR	95% CI^3^	*P* value	OR	95% CI	*P* value	OR	95% CI	*P* value
Female	1.3	0.9–1.7	0.146	1.2	1.0–1.5	0.092	1.3	0.9–1.7	0.175
Age > 60	1.0	0.7–1.5	0.941	1.2	0.9–1.6	0.318	1.1	0.7–1.6	0.833
Low education^2^	1.5	1.1–2.0	0.011	1.8	1.4–2.3	< 0.001	1.7	1.1–2.3	0.007
Non-native Norwegian speaker	NS	NS	NS	1.7	1.1–2.7	0.010	2.4	1.4–4.1	0.002
ASA^3^ grade > 2	NS	NS	NS	NS	NS	NS	2.6	1.5–4.8	0.002
Obesity^4^	1.8	1.3–2.6	0.001	NS	NS	NS	1.5	1.1–2.3	0.025
Smoking	1.9	1.4–2.6	< 0.001	1.6	1.3–2.1	0.001	1.6	1.1–2.3	0.008
Anxiety/depression^5^	1.5	1.1–2.1	0.009	1.5	1.2–1.8	0.001	1.4	1.0–2.0	0.041
Back pain > NRS^6^ 5	NS	NS	NS	1.5	1.1–2.0	0.015	3.0	1.3–2.7	0.009
Back pain > leg pain	NS	NS	NS	1.7	1.3–2.2	< 0.001	NS	NS	NS
Back pain > 12 months	2.3	1.8–3.1	< 0.001	2.4	1.9–3.0	< 0.001	2.8	2.0–3.9	< 0.001
Previously operated	1.9	1.3–2.8	< 0.001	2.3	1.8–3.0	< 0.001	1.9	1.4–2.7	0.009
Unresolved disability pension issue^7^	2.8	1.7–4.9	< 0.001	1.7	1.2–2.4	0.001	1.7	1.1–2.5	0.013
Unresolved insurance claim^8^	NS	NS	NS	1.6	1.0–2.5	0.048	1.7	1.0–3.0	0.048

^1^Range: 0*–*100 (no-maximal disability). The ODI score was < 33, 33*–*58, and > 58 in the subgroups with low, medium high baseline disability, respectively.^2^Less than 4 years of college/university education. ^3^American Society of Anesthesiologists grade. ^4^Body mass index ≥ 30. ^5^EQ-5D 3L questionnaire; 5^th^ item, moderate to severe problems. ^6^Numeric rating scale (0*–*10). ^7^Pending medical claim/litigation the Norwegian public welfare agency fund concerning disability pension. ^8^Pending medical compensation claim/litigation against private insurance companies or the public Norwegian System of Compensation to Patients

**Table 3:** Results from the multiple regression model showing associations (odds ratio (OR) and 95% confidence intervals (CI)) between predictors and patient-reported worsening (yes/no) after lumbar disc surgery, as defined by validated cut offs on the Oswestry Disability Index (ODI), split on subgroups with low, medium, and high baseline ODI scores (percentiles). For all predictors, except age and gender, NS indicates statistical insignificance, *p* value > 0.05

	OR for worsening by baseline ODI score^1^								
	Low ODI < 25^th^ percentile			Medium ODI 25^th^–75^th^ percentile			High ODI > 75^th^ percentile		
Predictor	OR^2^	95% CI	*P* value	OR	95% CI	*P* value	OR	95% CI	*P* value
Female	1.6	0.9–2.7	0.076	1.0	0.7–1.5	0.949	0.9	0.5–1.5	0.695
Age > 60	1.5	0.8–2.9	0.182	1.1	0.7–1.7	0.695	0.8	0.4–1.6	0.562
Low education^2^	2.7	1.5–5.1	0.002	1.8	1.1–2.7	0.010	2.0	1.1–3.7	0.022
Non-native Norwegian speaker	NS	NS	NS	2.8	1.6–4.9	0.001	3.8	1.9–7.6	< 0.001
ASA^3^ grade > 2	NS	NS	NS	NS	NS	NS	3.3	1.6–3.7	0.002
Obesity^4^	NS	NS	NS	NS	NS	NS	NS	NS	NS
Smoking	2.1	1.2–3.5	0.008	2.2	1.5–3.1	< 0.001	2.3	1.4–3.8	0.001
Anxiety/depression^5^	1.9	1.1–3.2	0.021	NS	NS	NS	NS	NS	NS
Back pain > NRS^6^ 5	NS	NS	NS	2.2	1.2–4.1	< 0.011	NS	NS	NS
Back pain > 12 months	2.7	1.6–4.5	< 0.001	2.9	2.0–4.2	< 0.001	3.4	2.1–5.6	< 0.001
Previously operated	2.6	1.4–4.6	0.002	3.3	2.3–4.8	< 0.001	NS	NS	NS
Unresolved insurance claim^7^	NS	NS	NS	NS	NS	NS	2.9	1.8–4.9	0.002

^1^Range: 0*–*100 (no-maximal disability) The ODI score was < 33, 33*–*58, and > 58 in the subgroups with low, medium high baseline disability, respectively. ^2^Less than 4 years of college/university education. ^3^American Society of Anesthesiologists grade. ^4^Body mass index ≥ 30. ^5^EQ-5D 3L questionnaire; 5^th^ item, moderate to severe problems. ^6^Numeric rating scale (0*–*10). ^7^Pending medical compensation claim/litigation against private insurance companies or the public Norwegian System of Compensation to Patients

**Fig. 3:**
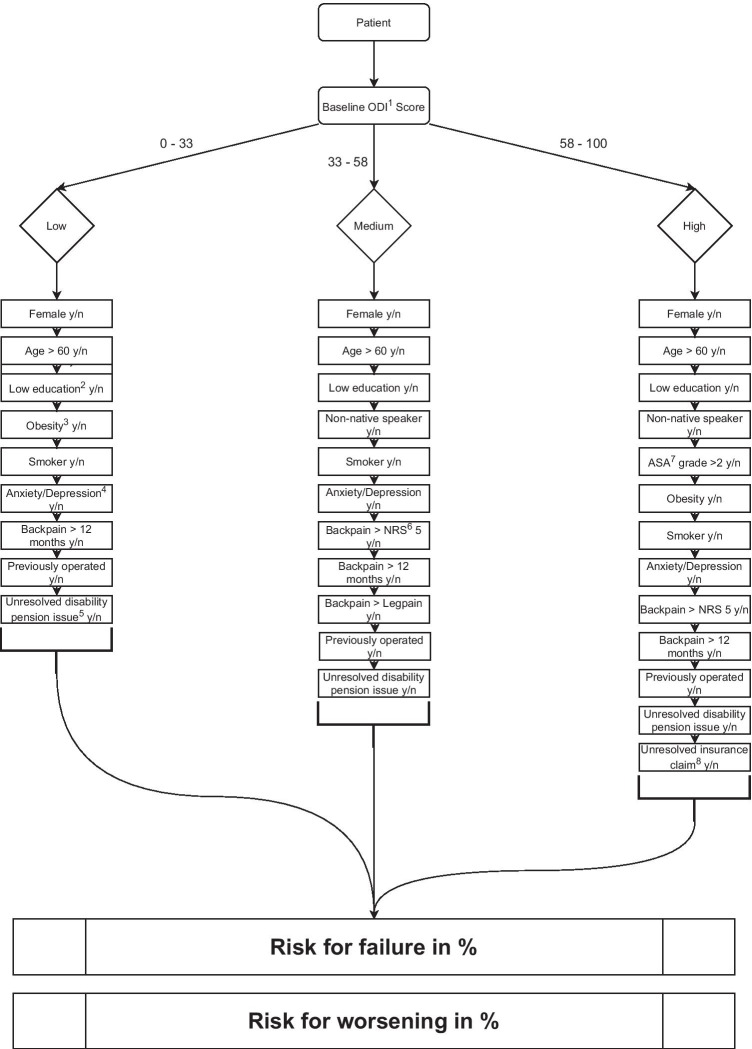
Model algorithm for the three ODI baseline groups. Based on the preoperative ODI the patient will be classified via one of the three pathways, calculating an overall risk for either failure or worsening. Risk is calculated from the odds of each risk factor. The risk factors are listed in random order, and their place in the sequence does not reflect their odds

The combination of the presence (yes) or absence (no) of each prognostic factor, as well as their respective odds ratios (Tables 2 and 3), yield an overall probability for failure or worsening in each of the three ODI baseline groups. The matrices are shown as a flow chart (Fig. 3). Table 4 illustrates three example cases from the risk matrices applied on the validation set. Each patient was allocated into 1 out of 6 matrices, based the baseline ODI (3 subgroups) and outcomes (2 subgroups). In the validation cohort, the individual predicted risk ranged from 3 to 94% for failure, and from 1 to 72% for worsening.

**Table 4:** Example cases from the validation set (patients 1*–*3) with different predicted probability (6 risk matrices) for failure and worsening based on baseline ODI score and presence (yes) or absence (no) of predictors. An open cell indicates that predictor was not relevant for the risk matrix the patient was assigned to

	Patient 1	Patient 2	Patient 3
Preoperative ODI score^1^	32	53	68
Female	No	Yes	No
Age > 60	Yes	Yes	Yes
Low education^2^	No	Yes	Yes
Non-native Norwegian speaker		No	No
ASA^3^ grade > 2			Yes
Obesity^4^	No		No
Smoking	No		Yes
Anxiety/depression^5^	Yes	Yes	Yes
Back pain > NRS^6^ 5		Yes	Yes
Back pain > leg pain		No	
Back pain > 12 months	No	Yes	Yes
Previously operated	No	No	Yes
Unresolved disability pension issue^7^	No	Yes	Yes
Unresolved insurance claim^8^		No	Yes
Predicted risk for failure	13%	50%	94%
Predicted risk for worsening	2%	6%	55%

^1^Range: 0*–*100 (no-maximal disability). ^2^Less than 4 years of college/university education. ^3^American Society of Anesthesiologists grade. ^4^Body mass index ≥ 30. ^5^EQ-5D 3 L questionnaire; 5^th^ item, moderate to severe problems. ^6^Numeric rating scale (0*–*10). ^7^Pending medical claim/ litigation the Norwegian public welfare agency fund concerning disability pension. ^8^Pending medical compensation claim/litigation against private insurance companies or the public Norwegian System of Compensation to Patients

The calibration plots showing agreement between the average predicted and observed proportion of failure and worsening (Fig. 2) illustrate that the predicted and observed probabilities coincided well. There was no statistically significant deviation of the coordinates from the optimal prediction line, except for the model predicting worsening in the >75^th^ percentile ODI baseline group.

**Fig. 4:**
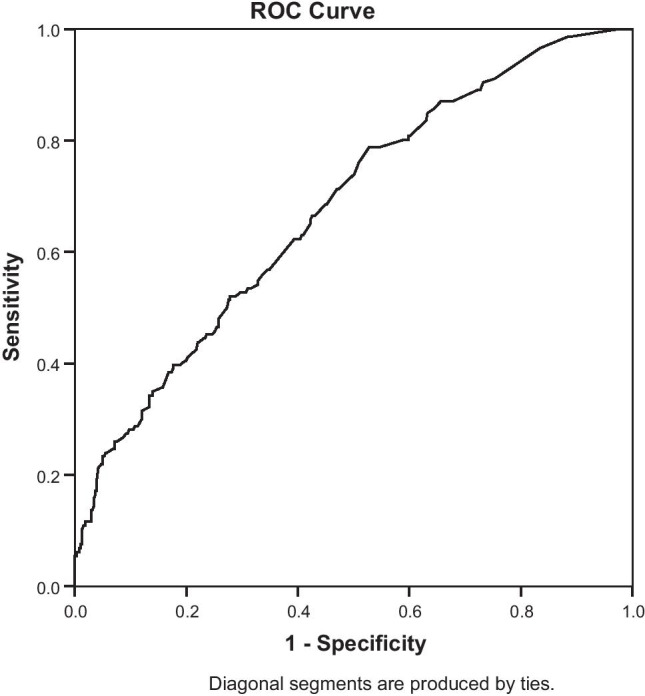
ROC (Receiver operating characteristics) analysis of predicted risk for failure vs not in the low baseline group (ODI < 25^th^ percentile). AUC (area under the curve, C-criterion) = 0.68, 95% CI=0.63–0.73

**Fig. 5:**
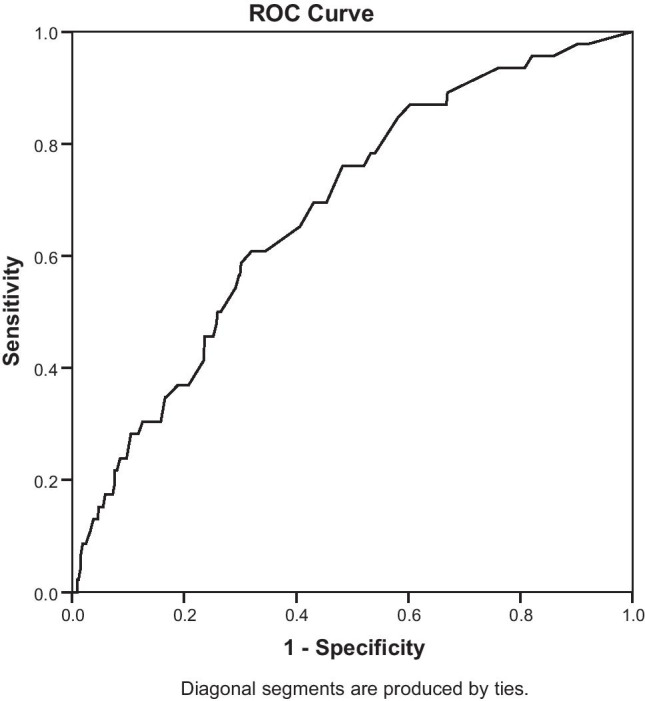
ROC (Receiver operating characteristics) analysis of predicted risk for worsening vs not in the high baseline group (ODI < 25^th^ percentile). AUC (area under the curve, C-criterion)=0.68, 95% CI=0.60–0.77

**Fig. 6:**
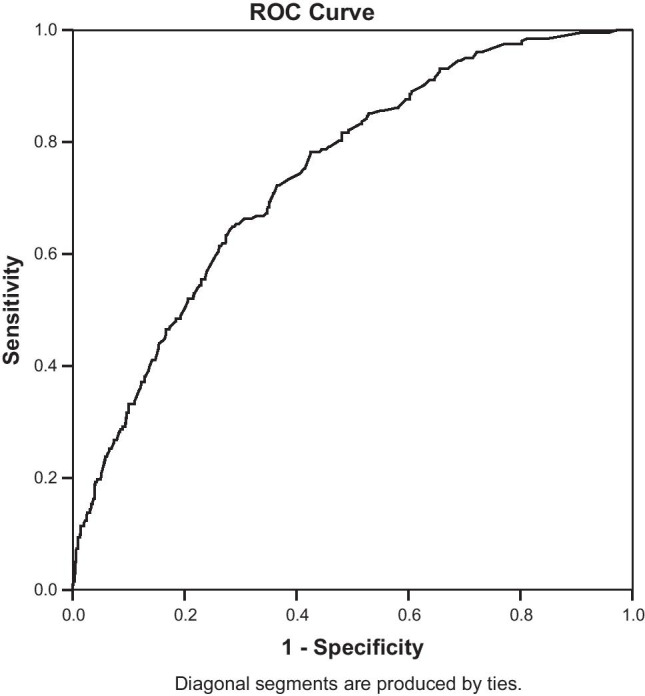
ROC (Receiver operating characteristics) analysis of predicted risk for failure vs not in the medium baseline group (ODI 25^th^-75^th^ percentile). AUC (area under the curve, C-criterion) = 0.74, 95% CI = 0.70–0.78

**Fig. 7:**
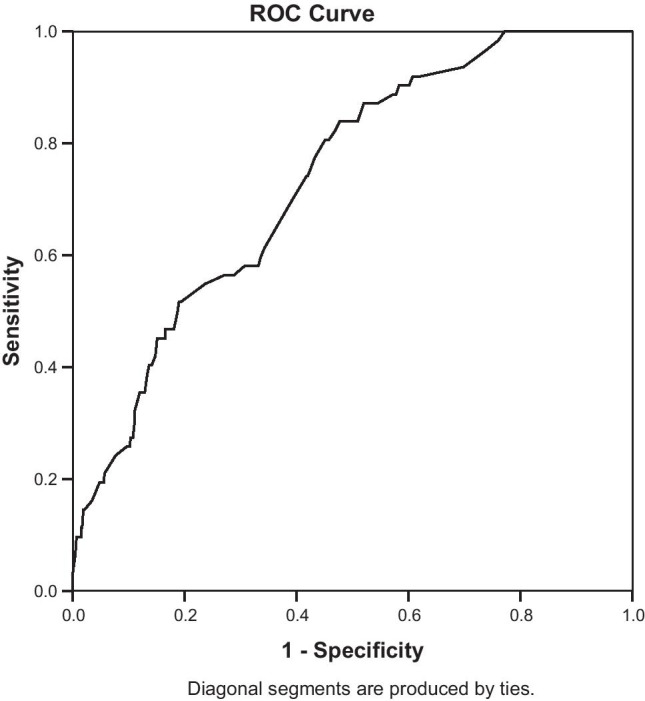
ROC (Receiver operating characteristics) analysis of predicted risk for worsening vs not in the medium baseline group (ODI 25^th^-75th percentile). AUC (area under the curve, C-criterion) = 0.74, 95% CI = 0.68–0.79

**Fig. 8:**
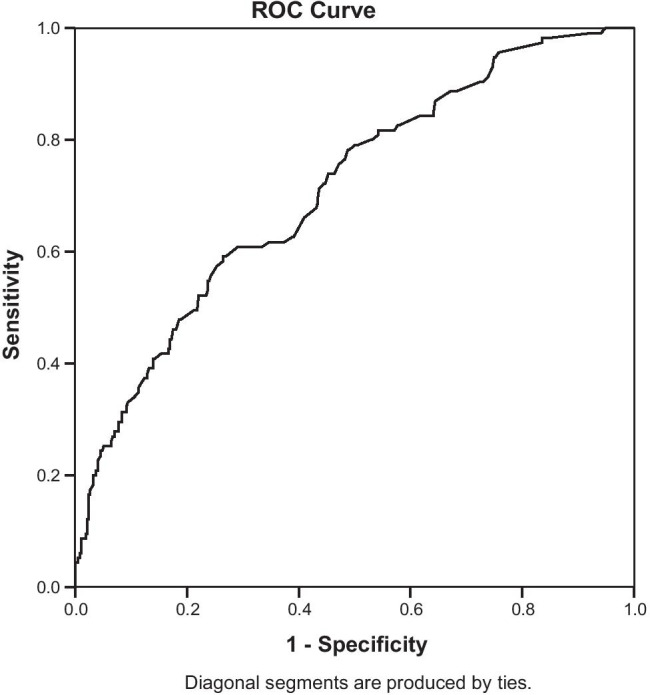
ROC (Receiver operating characteristics) analysis of predicted risk for failure vs not in the high baseline group (ODI > 75^th^ percentile). AUC (area under the curve, C-criterion) = 0.71, 95% CI = 0.66–0.76

**Fig. 9:**
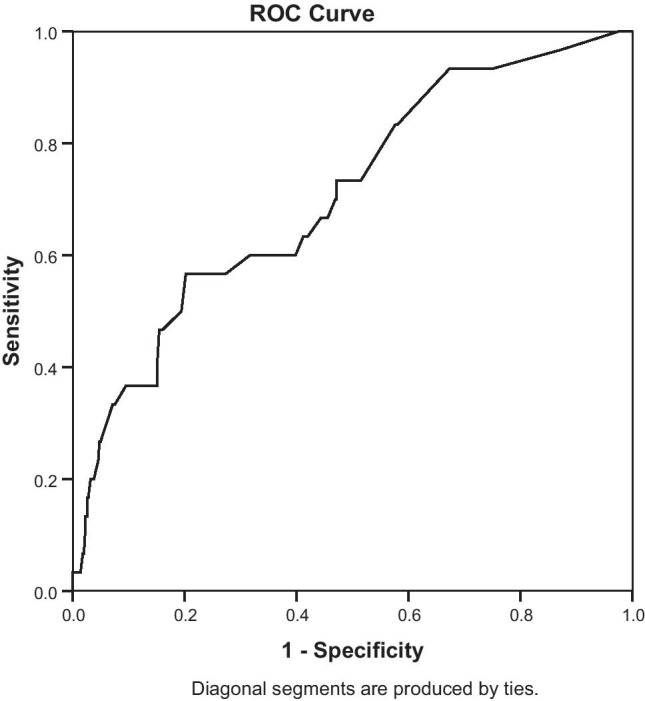
ROC (Receiver operating characteristics) analysis of predicted risk for worsening vs not in the high baseline group (ODI > 75^th^ percentile). AUC (area under the curve, C-criterion)=0.71, 95% CI=0.61–0.81

C-criterion values (95% CI) were 0.68 (0.63–0.73), 0.74 (0.70–0.78), and 0.71 (0.66–0.76) for prediction of failure in the low, medium, and high baseline ODI groups, respectively, indicating acceptable discrimination. The corresponding c-criterion values for predicting worsening were similar: 0.68 (0.60–0.76), 0.74 (0.68–0.79), and 0.71 (0.61–0.81). All ROC curves for C calculations are shown in the supplementary appendix (Figs. 4, 5, 6, 7, 8 and 9).

## Discussion

We have developed a prognostic model for unfavorable outcomes 12 months after surgery for lumbar disc herniation, based on validated and recommended PROMs [5]. The model can identify patients with a high and low baseline probability for those outcomes. Patients with low, medium, and high baseline ODI scores were associated with different sets of prognostic factors. Each factor has a different impact on the probability, shown as odds ratios in Tables 2 and 3. Higher odds ratios indicate higher probability for the outcome. The estimated preoperative probabilities in our study population ranged from 3% to 94% for failure and from 1% to 72% for worsening, exemplified by three cases. The model can be presented to surgeons and patients as a risk calculator, to facilitate individualized treatment recommendations.

It is important to acknowledge the conceptual differences between prognostic modeling and prognostic factors research. The prognostic model, developed in our study, aims at calculating the overall probability (individual absolute risk) for an outcome. Our study was not designed for prognostic factor research, which focuses on identifying independent prognostic (risk) factors [30, 34]. Still, our results can lend support to previously studies identifying a long duration of low back pain and leg pain, anxiety and/or depression, previous back surgery, smoking, lower education, BMI, and unresolved disability pension or insurance issues as predictors for inferior outcomes[12, 15, 17, 18, 29].

Prediction models have to balance the need for accurate predictions against the risk of overfitting. Model overfitting implies lack of generalizability, i.e., it might work well for the population it was developed on, but not for others [26]. For instance, it is important not to include too many and/or too specific covariates. Our model appeared to be well balanced between an acceptable accuracy and a limited number of predictors, which are available in most clinical trials and regular clinical practice at the hospitals. We stratified our model by different levels of baseline disability (low, medium, and high ODI score), since the outcome score is highly dependent on the baseline score, and the actual cut offs for failure and worsening are different in these subgroups [16, 18, 38].

The discriminative ability of risk the matrices was acceptable. Calibration assessment showed that for patients with high baseline disability (>75^th^ percentile of ODI) the model tended to underestimate the proportion of worsening, and the prediction of worsening among those cases was too inaccurate. A reason could be the small sample size (type II error) of this subgroup, or confounding due to unmeasured factors, such as widespread body pain and pain interference [1]. Confounding is the most likely source of bias in our study. We assessed anxiety and depression using one item of the EQ-5D 3L questionnaire, instead of a condition specific questionnaire which could be more sensitive. This may represent an information bias [12].

All cases of lumbar disc herniation were verified on MRI scans, evaluated by radiologists and surgeons. However, we did not have data on more specific morphological changes, e.g., contained versus uncontained herniation or additional Modic changes, which could influence the surgeon’s recommendation about surgery. This illustrates that statistical probabilities cannot be used as surrogate for clinical judgement, but rather as a supplementary decision support. We suggest that our model could be used in cases where the indication for surgery is uncertain. The model could be also helpful in calibrating surgeons’ and patients’ expectations about surgical outcomes.

To the best of our knowledge, this is the first registry study modeling unfavorable patient-reported outcomes after lumbar disc surgery. Three American studies have assessed patient populations operated for different degenerative spine disorders, including disc replacement and arthrodesis surgery [16, 24, 25]. The models were developed for predicting improvements, such as minimal clinically important change (MCIC), rather than unfavorable outcomes. Interestingly, 12 months of follow-up data from the latter paper by Khor et al. on a subgroup of 528 surgical patients showed that 222 of them reported an unsuccessful outcome (not reaching MCIC on the ODI scale) [16]. Of these, 86 (39%) reported to be unchanged or worse. The remaining 136 (61%) did not, hence representing a “grey zone” of patients with minor improvements. This supports our strategy of distinguishing failed from non-successful outcomes [38, 39].

Registry-based studies collecting “real-life” data from daily clinical practice have advantages such as large sample sizes and high external validity, but also limitations such as lower follow-up rates [11]. Loss to follow-up at 12 months was 32.7%. Baseline characteristics-linked inferior outcomes seemed to be equally distributed between responders and non-responders. Still, loss to follow-up could represent a selection bias, especially when estimating exact failure and worsening rates. However, two Scandinavian registry studies on similar patient populations found that loss to follow-up did not bias conclusions about treatment effects [13, 33]. Moreover, the objective of our study was not effectiveness evaluations, but rather to develop a prediction model over a wide range of outcomes.

The model should be externally validated in other cohorts, and its feasibility should be confirmed by patients and clinicians before being implemented in regular clinical practice. Importantly, we have not assessed outcomes after non-operative treatment. Therefore, it is highly uncertain if the model could be useful in other settings, e.g., among patients seen in general practice.

## Conclusion

We have developed a prognostic model to identify patients at risk of unfavorable outcomes after lumbar microdiscectomy, which could assist physicians and patients in clinical decision-making prior to surgery in cases where the indication for surgery is not clear cut. The model accounts for patients with different levels of preoperative disability and corresponding prognostic factors, facilitating individual based treatment recommendations.

## Appendix

**Table 5:** Failure and worsening 12 months after surgery for subgroups of different baseline disability (low, medium and high percentiles of the ODI score) in the training (n = 5741, 70%) and validation (n = 2218, 30%) set

	Training set			Validation set		
	ODI group^1^ < 25^th^	ODI group 25-75^th^	ODI group > 75^th^	ODI group < 25^th^	ODI group 25-75^th^	ODI group > 75^th^
Cases n (%)	1243 (24)	2772 (54)	1159 (22)	608 (27)	1024 (46)	586 (26)
Failure n (%)	366 (26)	565 (23)	306 (23)	165 (27)	229 (22)	148 (25)
Worsening n (%)	76 (5)	157 (7)	85 (7)	50 (8)	65 (6)	36 (6)

^1^Baseline ODI group based on the baseline percentile of the ODI score – low (<25^th^ percentile, <33 points), medium (25^th^ – 75^th^ percentile, 33-58 points), high (>75^th^ percentile, >58 points). ODI range: 0-100 (no-maximal disability)

**Table 6:** Results from the univariate binary logistic regression analyses of failure in both the training (n = 5741) and validation (n = 2218) cohort, showing associations (Odds Ratio (OR) and 95% confidence intervals (CI)) between predictors and patient reported "failure" (unchanged or worse, yes/no) of lumbar disc surgery, as defined by validated cut offs on the Oswestry Disability Index (ODI), split by subgroups with low, medium and high baseline ODI scores (percentiles). For all predictors, except age and gender, NS indicates statistical insignificance, p value > 0.1

	Training Cohort						Validation Cohort					
Baseline ODI^1^ group	< 25^th^		25^th^-75^th^		> 75^th^		< 25^th^		25^th^-75^th^		> 75^th^	
Predictor	OR^2^	P value	OR	P value	OR	P value	OR	P value	OR	P value	OR	P value
Age > 60	1.0 (0.7 – 1.4)	0.986	1.4 (1.2 – 1.8)	0.002	1.7 (1.2 – 2.2)	0.001	1.2 (0.7 – 1.9)	0.526	1.5 (1.1 – 2.1)	0.020	0.9 (0.6 – 1.4)	0.498
Living alone	NS^3^	0.488	NS	0.347	1.5 (1.1 – 2.0)	0.011	0.5 (0.3 – 0.9)	0.009	NS	0.213	1.7 (1.1 – 2.5)	0.014
Nonnative Norwegian speaker	1.9 (1.1- 3.5)	0.030	2.1 (1.5 – 3.0)	< 0.001	2.3 (1.5 – 3.6)	< 0.001	NS	0.415	1.9 (1.1 – 3.2)	0.027	2.2 (1.2 – 4.1)	0.015
Female	1.2 (0.9 – 1.6)	0.147	1.2 (1.0 – 1.4)	0.050	1.5 (1.2 – 2.0)	0.002	1.2 (0.8 – 1.7)	0.454	1.1 (0.8 – 1.5)	0.467	1.4 (0.9 – 2.0)	0.106
Smoking	2.3 (1.7 – 3.0)	< 0.001	1.9 (1.6 – 2.3)	< 0.001	2.0 (1.5 – 2.6)	< 0.001	1.5 (1.0 – 2.3)	0.046	1.6 (1.2 – 2.2)	0.003	1.7 (1.1 – 2.6)	0.012
Low education^2^	2.0 (1.5 – 2.6)	< 0.001	2.1 (1.7 – 2.5)	< 0.001	2.3 (1.7 – 3.0)	< 0.001	2.0 (1.4 – 3.0)	< 0.001	2.3 (1.7 – 3.3)	< 0.001	1.8 (1.2 –2.8)	0.003
Obesity^3^	1.9 (1.4 – 2.6)	< 0.001	1.2 (1.0 – 1.5)	0.096	2.0 (1.5 – 2.7)	< 0.001	NS	0.211	1.7 (1.2 – 2.5)	0.003	2.5 (1.5 – 4.0)	< 0.001
ASA^4^ grade 2	NS	0.134	2.0 (1.5 – 2.8)	< 0.001	4.3 (2.8 – 6.6)	< 0.001	NS	0.264	2.5 (1.5 – 4.3)	0.001	2.5 (1.3 – 4.6)	0.005
Diabetes Mellitus	2.2 (1.1 – 4.7)	0.043	NS	0.149	4.0 (2.2 – 7.1)	< 0.001	NS	0.446	2.2 (1.2 – 4.1)	< 0.013	4.5 (1.7 – 12.0)	0.003
Anxiety/Depression	1.8 (1.5 – 2.5)	< 0.001	1.8 (1.5 – 2.2)	< 0.001	2.0 (1.4 – 2.5)	< 0.001	1.8 (1.2 – 2.6)	0.004	1.9 (1.4 – 2.6)	< 0.001	NS	0.161
Unresolved disability pension issue^6^	4.8 (3.0 – 7.6)	< 0.001	3.1 (2.4 – 4.0)	< 0.001	3.5 (2.6 – 4.7)	< 0.001	2.8 (1.4 – 5.3)	0.002	3.3 (2.3 – 4.8)	< 0.001	5.1 (3.2 – 8.1)	< 0.001
Unresolved insurance claim^7^	1.9 (1.2 – 3.2)	0.011	2.0 (1.4 – 2.3)	< 0.001	2.3 (1.5 – 3.5)	< 0.001	2.2 (1.0 – 4.6)	0.042	2.0 (1.1 – 3.6)	0.019	3.3 (1.7 – 6.4)	< 0.001
Back pain > 12 months	2.8 (2.2 – 3.6)	< 0.001	2.6 (2.1 – 3.1)	< 0.001	3.8 (2.9 – 5.1)	< 0.001	2.2 (1.5 – 3.1)	< 0.001	3.3 (2.4 – 4.5)	< 0.001	2.0 (1.3 – 3.1)	0.001
Back pain worse than leg pain^8^	1.4 (1.0 – 1.8)	0.026	1.8 (1.5 – 2.3)	< 0.001	1.5 (1.1 – 2.2)	0.015	NS	0.842	1.5 (1.0 – 2.1)	0.035	1.6 (1.0 – 2.7)	0.067
Paresis < grade 4	NS	0.160	NS	0.588	NS	0.565	NS	0.403	NS	0.782	NS	0.102
Previously operated	2.0 (1.4 – 2.7)	< 0.001	2.2 (1.8 – 2.7)	< 0.001	1.9 (1.4 – 2.5)	< 0.001	2.5 (1.6 – 3.8)	< 0.001	2.1 (1.5 – 2.9)	< 0.001	2.3 (1.5 – 3.4)	0.001
> 2 previous surgeries	4.8 (1.2 – 20.3)	0.032	4.6 (1.8 – 11.6)	0.001	3.8 (1.5 – 9.9)	0.007	NS	0.169	2.9 (0.9 – 9.7)	0.078	7.0 (2.1 – 23.2)	0.001
Back pain > NRS^8^ 5	1.6 (1.2 – 2.0)	< 0.001	2.2 (1.8 – 2.8)	< 0.001	4.4 (2.3 – 8.5)	< 0.001	2.0 (1.3 – 2.9)	< 0.001	2.7 (1.7 – 4.3)	< 0.001	2.6 (1.2 – 5.6)	0.014
Leg pain > NRS 5	NS	0.911	NS	0.111	NS	0.387	NS	0.205	NS	0.757	NS	0.701
Daily use of analgesics	0.8 (0.6 – 1.0)	0.030	NS	0.932	NS	0.172	0.6 (0.4 – 1.9)	0.015	NS	0.165	NS	0.914

^1^Range: 0-100 (no-maximal disability). The ODI score was <33, 33-58, and >58 in the subgroups with low, medium high baseline disability.^2^Less than four years of college/university education. ^3^Body Mass Index ≥30. ^4^American Society of Anesthesiologists grade. ^5^EQ-5D 3L questionnaire; 5^th^ item, moderate to severe problems. ^6^Pending medical claim/litigation with the Norwegian public welfare agency fund concerning disability pension. ^7^Pending medical compensation claim/litigation against private insurance companies or the public Norwegian System of Compensation to Patients. ^8^Numeric Rating Scale (0-10)

**Table 7:** Results from the univariate binary logistic regression analyses of worsening in both the training (n =5741) and validation (n =2218) cohort, showing associations (Odds Ratio (OR) and 95% confidence intervals (CI)) between predictors and patient reported worsening (yes/no) of lumbar disc surgery, as defined by validated cut offs on the Oswestry Disability Index (ODI), split on subgroups with low, medium and high baseline ODI scores (percentiles). For all predictors, except age and gender, NS indicates statistical insignificance, p value > 0.1

	Training Cohort						Validation Cohort					
Baseline ODI^1^ group	< 25^th^		25^th^-75^th^		> 75^th^		< 25^th^		25^th^-75^th^		> 75^th^	
Predictor	OR	P value	OR	P value	OR	P value	OR	P value	OR	P value	OR	P value
Age > 60	1.4 (0.8 – 2.5)	0.233	1.1 (0.8 – 1.7)	0.554	1.3 (0.8 – 2.2)	0.304	1.7 (0.8 – 3.3)	0.146	1.7 (1.0 – 2.9)	0.067	0.9 (0.4 – 2.0)	0.748
Living alone	1.8 (1.1 – 2.9)	0.028	NS^3^	0.389	NS	0.114	NS	0.506	NS	0.962	NS	0.264
Nonnative Norwegian speaker	3.2 (1.4 – 7.4)	0.006	2.6 (1.6 – 4.3)	< 0.001	3.5 (1.9 – 6.3)	< 0.001	NS	0.159	3.6 (1.8 – 7.4)	< 0.001	NS	0.401
Female	1.5 (0.9 – 2.4)	0.097	1.0 (0.7 – 1.3)	0.754	1.1 (0.7 – 1.7)	0.640	1.3 (1 – 1.8)	0.104	1.3 (0.8 – 2.1)	0.360	0.6 (0.3 – 1.3)	0.185
Smoking	3.1 (1.9 – 5.0)	< 0.001	2.5 (1.8 – 3.4)	< 0.001	2.7 (1.7 – 4.2)	< 0.001	2.4 (1.3 – 4.3)	0.006	1.8 (1.1 – 3.0)	0.029	NS	0.390
Low education^2^	3.9 (2.1 – 7.1)	< 0.001	2.3 (1.5 – 3.3)	< 0.001	2.7 (1.5 – 4.6)	< 0.001	2.7 (1.3 – 5.5)	0.007	2.6 (1.4 – 4.9)	0.003	NS	0.550
Obesity^3^	NS	0.277	NS	0.184	1.8 (1.1 – 3.0)	0.024	NS	0.302	NS	0.832	NS	0.145
ASA^4^ grade 2	2.5 (1.0 – 6.7)	0.058	NS	0.146	3.1 (1.7 – 5.7)	< 0.001	NS	0.105	2.4 (1.1 – 5.2)	0.033	2.7 (1.0 – 6.8)	0.041
Diabetes Mellitus	NS	0.216	1.9 (0.9 – 3.9)	0.081	2.5 (1.1 – 5.8)	0.029	NS	0.592	NS	0.477	NS	0.954
Anxiety/Depression	2.4 (1.5 – 3.9)	< 0.001	1.6 (1.2 – 2.2)	0.005	1.9 (1.2 – 3.1)	0.012	NS	0.849	3.1 (1.8 – 5.3)	< 0.001	NS	0.253
Unresolved disability pension issue^6^	3.8 (2.0 – 7.3)	< 0.001	2.1 (1.4 – 3.2)	< 0.001	2.7 (1.7 – 4.3)	< 0.001	3.3 (1.4 – 7.6)	0.005	3.3 (2.3 – 4.8)	< 0.001	5.5 (2.7 – 11.3)	< 0.001
Unresolved insurance claim^7^	NS	0.407	1.8 (1.2 – 2.6)	0.002	3.8 (2.1 – 6.9)	< 0.001	3.8 (1.6 – 9.4)	0.004	NS	0.736	3.5 (1.4 – 9.2)	0.009
Back pain > 12 months	3.1 (1.8 – 5.1)	< 0.001	3.3 (2.4 – 4.7)	< 0.001	4.4 (2.8 – 7.0)	< 0.001	NS	0.147	3.9 (2.3 – 6.6)	< 0.001	5.1 (2.5 – 10.5)	< 0.001
Back pain worse than leg pain^8^	NS	0.129	NS	0.121	NS	0.561	NS	0.490	NS	0.27	NS	0.519
Paresis < grade 4	NS	0.939	NS	0.588	NS	0.290	NS	0.143	NS	0.809	NS	0.657
Previously operated	2.4 (1.4 – 4.1)	0.001	3.0 (2.2 – 4.2)	< 0.001	1.5 (0.9 – 2.4)	0.099	2.1 (1.1 – 4.0)	0.023	2.2 (1.3 – 3.8)	0.002	3.3 (1.7 – 6.6)	0.001
> 2 previous surgeries	11.0 (2.6 – 46.8)	0.001	3.693	0.021	3.2 (0.9 – 11.3)	0.074	NS	0.159	NS	0.127	11 (3.4 – 35.4)	< 0.001
Back pain > NRS^8^ 5	2.583	< 0.001	3.044	< 0.001	5.3 (1.3 – 21.8)	0.021	2.9 (1.4 – 6.0)	0.003	5.3 (1.7 – 17.1)	0.005	NS	0.997
Leg pain > NRS 5	1.544	0.098	NS	0.137	NS	0.998	1.8 (0.9 – 3.7)	0.870	NS	0.159	NS	0.999
Daily use of analgesics	NS	0.841	NS	0.678	NS	0.437	0.4 (0.2 – 0.7)	0.002	NS	0.595	NS	0.998

^1^Range: 0-100 (no-maximal disability). The ODI score was <33, 33-58, and >58 in the subgroups with low, medium high baseline disability.^2^Less than four years of college/university education. ^3^Body Mass Index ≥30. ^4^American Society of Anesthesiologists grade. ^5^EQ-5D 3L questionnaire; 5^th^ item, moderate to severe problems. ^6^Pending medical claim/litigation with the Norwegian public welfare agency fund concerning disability pension. ^7^Pending medical compensation claim/litigation against private insurance companies or the public Norwegian System of Compensation to Patients. ^8^Numeric Rating Scale (0-10)
